# Total Hip and Knee Arthroplasty Implant Revision Risk to 5 Years From a State-wide Arthroplasty Registry in Michigan

**DOI:** 10.1016/j.artd.2023.101146

**Published:** 2023-05-23

**Authors:** Richard E. Hughes, Huiyong Zheng, Tae Kim, Brian R. Hallstrom

**Affiliations:** Department of Orthopaedic Surgery, University of Michigan, Ann Arbor, MI, USA

**Keywords:** Arthroplasty, Hip, Knee, Implants, Registry, Revision

## Abstract

**Background:**

Information on the revision risk of implants is useful for improving the quality of care for elective hip and knee arthroplasty. The purpose of this study was to report on the revision risk of implants using a state-wide registry in the United States.

**Methods:**

The Michigan Arthroplasty Registry Collaborative Quality Initiative systematically collects data on elective primary and revision hip and knee arthroplasty cases in Michigan. It contained data on 139,970 hip and 245,499 knee arthroplasty cases from February 15, 2012, to December 31, 2021. Kaplan-Meier estimates of revision risk were computed using time to first revision as the dependent variable, and the results were computed and expressed as the cumulative percent revision (CPR). CPR estimates were computed for all implants having at least 500 cases in the Michigan Arthroplasty Registry Collaborative Quality Initiative dataset.

**Results:**

At 5-years postoperatively, elective primary conventional total hip arthroplasty implant stem/cup combinations had CPR values from 0.95% (0.39%-2.30%, 95% confidence intervals [CI]) to 5.77% (4.22%-7.85%, 95% CI), and elective primary total knee arthroplasty CPR ranged from 1.10% (0.64%-1.89%, 95% CI) to 12.52% (8.37%-18.50%, 95% CI). Unicondylar knee arthroplasty CPR at 5-years went from 4.23% (3.54%-5.06%, 95% CI) to 7.13% (6.20%-8.20%, 95% CI).

**Conclusions:**

The wide variation in CPR points to the need for surgeons to choose implants wisely to improve quality of care.

## Introduction

Surgeons choose implants for hip and knee replacements based on their training, experience, mentors, marketing, and availability at their hospital. Hospitals choose implants based on cost, contracts, and other business considerations. Rarely, the performance of the implants themselves is part of the conversation. In 2018, there were 715,203 and 599,494 hip and knee replacements in the United States, respectively [1]. One consequence of the very large number of procedures is that even a statistically rare event affects the lives of many patients. While hip and knee arthroplasty is relatively safe, it carries the risk of complications and revision surgery. In Michigan, for example, the 5-year risk of revision for elective primary hip and knee replacements was 2.7% and 3.0%, respectively [2].

There is substantial variation in revision risk across implants, and this creates an opportunity for surgeons and hospitals to reduce the number of revisions by selecting implants that have a lower revision risk. In a review of arthroplasty registry data for total hip arthroplasty (THA) implants, Hughes *et al.* [3] found the revision risk at 10-years postoperatively to range between 1.03% and 66.5%. In a similar review of arthroplasty registry data for knees, Foster *et al.* [4] found a range of 2.4% to 35.7% for cemented implants. National and regional registries outside the United States have long produced publicly available reports that provide revision risk data by implant product name and/or brand. However, such data in the United States is scarce. The American Joint Replacement Registry (AJRR) reports revision risk by implant based on a dataset that is merged with Medicare claims data, so their reported revision risk is limited to patients 65 years of age and older. Neither the Kaiser Permanente National Total Joint Replacement Registry [5] nor the Function and Outcomes Research for Comparative Effectiveness in Total Joint Replacement registry [6] publicly reports the revision risk of implants. However, the Michigan Arthroplasty Registry Collaborative Quality Initiative (MARCQI) does produce a publicly available annual report that gives revision risk data up to 5 years postoperatively. MARCQI is a state-wide quality improvement collaborative in Michigan [[7], [8], [9]].

The purpose of this project was to quantify the variation in 1- and 5-year revision risks of THA and total knee arthroplasty (TKA) implants in a regional arthroplasty registry in the United States. The hypothesis was that 1- and 5-year revision risk varied across implant designs for total hip and knee arthroplasty. The study was based on the 2022 MARCQI annual report [2].

## Material and methods

All analyses were based on the patient registry developed and maintained by MARCQI. MARCQI is a state-wide quality improvement collaborative [[7], [8], [9]]. It consists of 76 participating facilities (64 hospitals, 4 hospital outpatient departments, and 8 ambulatory surgery centers). MARCQI operates under a determination of “not regulated” status (45 Code of Federal Regulations, CFR 46) by the University of Michigan’s Institutional Review Board due to its mission of quality improvement. It collects data on 97% of all elective total hip and knee arthroplasty cases performed in the state of Michigan. Trained data abstractors are employed at each hospital to enter data into the MARCQI patient registry through a combination of an online database portal and a file-based upload. Data collected includes case details, patient demographics, patient comorbidities, and adverse events that occur within 90 days of surgery. Moreover, in order to analyze revision risk, the dates of primary and revision cases are captured, and they are linked based on a patient identifier, joint (hip or knee), and laterality (right or left), as well as the date/time on which the case was performed. It is important to note that MARCQI can link revision cases that occur at a different hospital from where the primary procedure was performed, unless either is performed out-of-state or at one of the few hospitals in Michigan that do not participate in MARCQI. The catalog numbers of all implanted devices for each case are also recorded in the patient registry.

Because MARCQI seeks to report revision risk data by implant product name in its annual report, catalog numbers must be converted to product names. This was done using a “device library,” which serves as a mapping from catalog number to device characteristics [10]. The library used for this work was created by Mendenhall Associates Inc. and is now owned by Curvo Labs. In addition to the implant product name, the library can also provide information about component characteristics such as knee constraint, implant dimensions, and materials. While the full 2022 MARCQI annual report contains more details, this paper focuses on analysis by product name.

This analysis is based on all cases entered into the MARCQI database between its inception on February 15, 2012, and December 31, 2021. However, revision risk estimates were only performed for implant combinations and individual implants that had at least 500 cases in the database to assure reliable revision risk estimates.

The endpoint of interest was the first revision following the primary procedure. The time-to-revision for the first revision following the primary procedure was computed from the dates of the primary and revision cases. When no revision was recorded for a primary case by December 31, 2021, it was treated as censored in the survival analysis. The cumulative percent revision (CPR) for each implant product name combination and isolated implant was computed using a Kaplan-Meier estimator, provided there were at least 500 cases in the database. The Kaplan-Meier method produced a survivorship function, *S(t)*, and the CPR is 100∗(1-*S(t)*). At any point in time, *t*, the CPR is the percentage of cases that have been revised at or before *t* following the primary procedure. 95% confidence intervals (CI) were computed. Cox proportional hazards models were fit to the survival data, adjusting for sex and age. The 90-day death rate without any event(s) after primary surgery was approximately 0.18% and was excluded from the survival analysis. For a detailed description of statistical methods, refer to Appendix A in reference [2].

All statistical analyses were performed using SAS (Version 9.4, SAS Institute, Cary, NC).

## Results

**Figure 1:**
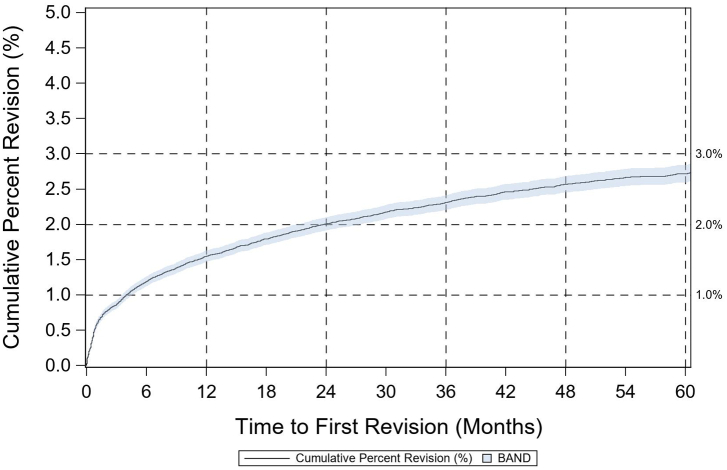
Cumulative percent revision for primary conventional THA.

The dataset contained 139,970 THA cases. 92.1% were primary cases, and 7.9% were revisions. There are 1170 hip resurfacing cases. The mean and standard deviation (SD) of age of the patients were 65.2 (SD 11.2), and the mean body mass index (BMI) was 30.5 (SD 6.4). 54.6% were female. For primary THA cases, the mean age was 65.0 (SD 11.1) and the mean BMI was 30.6 (SD 6.3). 54.5% were female. The distribution of approaches used in primary THA was 30.9% anterior, 22.2% anterolateral, 45.6% posterior, 0.5% transtrochanteric, and 0.8% other/missing. 85.4% of the cases were performed for a diagnosis of osteoarthritis. 96.8% were conventional, 1.1% were resurfacing, and 2.1% were conversion cases. The CPR for all primary conventional THAs at 1 year was 1.55% (1.48%-1.62%, 95% CI) (Fig. 1) and it was 1.29% (0.78%-2.13%, 95% CI) for resurfacing THAs. At 5 years, the CPR was 2.66% (2.57%-2.77%, 95% CI) for conventional and 3.05% (2.14%-4.33%, 95% CI) for resurfacing.

There was a wide range of CPRs within both 1-year and 5-year data for primary THA (Table 1). At 1-year post-surgery, the Kaplan-Meier estimate of the CPR for cup-stem combinations ranged from 0.16% (0.02%-1.10%, 95% CI) to 3.73% (2.76%-5.03%, 95% CI). For cups, it went from 0.88% (0.37%-2.11%, 95% CI) to 2.11% (1.82%-2.44%, 95% CI), and stems varied from 0.15% (0.02%-1.09%, 95% CI) to 3.53% (2.72%-4.56%, 95% CI). At 5-years, the CPR ranged from 0.95% (0.39%-2.30%, 95% CI) to 5.77% (4.22%-7.85%, 95% CI) for stem-cup combinations. For stems at 5 years, it went from 0.95% (0.39%-2.29%, 95% CI) to 5.07% (4.04%-6.36%, 95% CI) (Table 2), and cups varied from 1.70% (0.89%-3.24%, 95% CI) to 3.82% (3.02%-4.83%, 95% CI) (Table 3).

**Table 1:** Stem-cup combinations, 1-year and 5-year CPR with hazard ratio (HR) and 95% confidence interval (CI).

Stem	Cup	Number of cases (N)	1-year CPR (95% CI)	5-year CPR (95% CI)	Implant HR (95% CI)
Accolade II	Trident	20,928	1.17 (1.03, 1.32)	2.34 (2.14, 2.57)	N/A^b^
Accolade II	Trident II	11,883	1.57 (1.35, 1.82)	2.07 (1.76, 2.44)	N/A^b^
Accolade TMZF	Trident	913	0.88 (0.44, 1.74)	2.74 (1.86, 4.03)	1.13 (0.74, 1.73)
Actis DuoFix	Pinnacle	4068	0.43 (0.26, 0.71)	N/A^a^	0.30 (0.19, 0.48)
AML	Pinnacle	761	1.34 (0.72, 2.48)	3.02 (1.91, 4.75)	0.93 (0.48, 1.82)
Anthology	PolarCup	542	0.56 (0.18, 1.72)	2.26 (1.19, 4.27)	0.44 (0.17, 1.17)
Anthology	Reflection 3	4226	2.13 (1.73, 2.63)	3.49 (2.91, 4.18)	1.36 (1.08, 1.72)
Avenir	G7	1380	0.70 (0.36, 1.34)	2.50 (0.61, 10.05)	0.53 (0.28, 1.00)
Avenir Muller	Continuum	530	1.32 (0.63, 2.76)	2.33 (1.33, 4.06)	1.06 (0.59, 1.91)
Avenir Muller	G7	580	1.92 (1.07, 3.44)	2.16 (1.23, 3.78)	N/A^b^
Corail	Pinnacle	3148	1.19 (0.86, 1.65)	1.97 (1.51, 2.57)	0.92 (0.67, 1.25)
Corail Coxa Vara	Pinnacle	665	0.16 (0.02, 1.10)	0.95 (0.39, 2.30)	0.38 (0.15, 0.91)
Echo Bi-Metric	G7	1549	2.74 (2.03, 3.69)	4.00 (3.03, 5.27)	N/A^b^
Echo Bi-Metric Microplasty	G7	1657	1.98 (1.41, 2.79)	3.25 (2.36, 4.47)	1.49 (1.05, 2.12)
Fitmore	Continuum	2817	1.32 (0.96, 1.81)	2.22 (1.73, 2.85)	1.29 (0.95, 1.74)
Fitmore	G7	2434	1.21 (0.84, 1.73)	1.97 (1.36, 2.84)	N/A^b^
M/L Taper^c^	Continuum	7767	1.70 (1.43, 2.02)	2.80 (2.44, 3.21)	0.97 (0.81, 1.17)
M/L Taper^c^	G7	2981	1.74 (1.32, 2.29)	2.60 (2.04, 3.31)	N/A^b^
M/L Taper^c^	Trabecular metal	1011	2.61 (1.78, 3.80)	3.84 (2.78, 5.31)	1.27 (0.75, 2.14)
M/L Taper^c^	Trilogy	1607	1.51 (1.01, 2.24)	3.09 (2.33, 4.10)	N/A^b^
Polarstem	Reflection 3	2261	1.55 (1.11, 2.18)	2.54 (1.81, 3.55)	1.03 (0.72, 1.47)
Secur-Fit	Trident	1099	3.73 (2.76, 5.03)	5.50 (4.28, 7.05)	1.84 (1.31, 2.59)
Secur-Fit	Trident II	515	3.06 (1.85, 5.03)	N/A^a^	N/A^b^
Secur-Fit Max	Trident	2898	1.95 (1.51, 2.53)	3.29 (2.67, 4.04)	1.11 (0.84, 1.49)
Secur-Fit Plus Max	Trident	2018	1.69 (1.21, 2.35)	2.52 (1.91, 3.31)	0.91 (0.67, 1.26)
SROM	Pinnacle	1103	1.27 (0.76, 2.14)	3.24 (2.32, 4.51)	N/A^b^
Summit	Pinnacle	6975	1.44 (1.18, 1.75)	2.08 (1.76, 2.46)	N/A^b^
Synergy	Reflection 3	1161	2.54 (1.77, 3.63)	4.04 (3.00, 5.43)	1.44 (0.99, 2.10)
Taperloc 133	Continuum	761	2.91 (1.93, 4.39)	5.77 (4.22, 7.85)	1.45 (0.97, 2.16)
Taperloc 133	G7	8494	1.72 (1.46, 2.03)	2.87 (2.43, 3.39)	1.03 (0.86, 1.25)
Taperloc 133	Regenerex RingLoc	501	1.60 (0.80, 3.17)	2.82 (1.68, 4.73)	0.87 (0.47, 1.61)
Taperloc 133	RingLoc+	1704	1.70 (1.19, 2.44)	2.63 (1.96, 3.52)	0.82 (0.58, 1.17)
Taperloc 133 Microplasty	G7	3101	1.18 (0.85, 1.64)	2.08 (1.52, 2.84)	0.75 (0.55, 1.02)
Trabecular Metal	Continuum	745	2.32 (1.45, 3.70)	3.23 (2.16, 4.83)	1.17 (0.70, 1.98)
Tri-Lock BPS	Pinnacle	3608	0.58 (0.37, 0.90)	1.51 (1.09, 2.10)	N/A^b^

a indicates zero patients at risk at 5 y following primary procedure.

b indicates the proportional hazards assumption of the Cox model was not met.

c indicates this product does not include M/L Taper Kinectiv.

**Table 2:** Femoral stems, 1-year and 5-year CPR with hazard ratio (HR) and 95% confidence interval (CI).

Stem	Number of cases (N)	1-year CPR (95% CI)	5-year CPR (95% CI)	Implant HR (95% CI)
Accolade C	628	1.00 (0.45, 2.21)	1.92 (0.96, 3.84)	0.74 (0.40, 1.39)
Accolade II	33,414	1.31 (1.19, 1.44)	2.45 (2.26, 2.65)	N/A^b^
Accolade TMZF	915	0.87 (0.44, 1.74)	2.73 (1.86, 4.02)	1.13 (0.74, 1.73)
Actis DuoFix	4168	0.50 (0.32, 0.78)	N/A^a^	0.34 (0.22, 0.53)
AML	764	1.34 (0.72, 2.47)	3.00 (1.90, 4.72)	0.92 (0.47, 1.80)
Anthology	4811	1.95 (1.59, 2.40)	3.39 (2.85, 4.03)	N/A^b^
Avenir	1481	0.81 (0.45, 1.46)	1.70 (0.59, 4.84)	0.55 (0.31, 0.99)
Avenir Muller	1132	1.79 (1.16, 2.77)	2.52 (1.71, 3.72)	1.21 (0.77, 1.90)
Corail	3158	1.22 (0.88, 1.68)	2.00 (1.53, 2.60)	0.94 (0.69, 1.28)
Corail Coxa Vara	670	0.15 (0.02, 1.09)	0.95 (0.39, 2.29)	0.37 (0.15, 0.91)
Echo Bi-Metric	1890	2.62 (1.98, 3.45)	3.74 (2.92, 4.79)	N/A^b^
Echo Bi-Metric Microplasty	1659	1.98 (1.40, 2.79)	3.24 (2.35, 4.46)	1.49 (1.05, 2.12)
Fitmore	5657	1.25 (0.99, 1.58)	2.18 (1.81, 2.63)	1.10 (0.84, 1.43)
Integral X	552	2.57 (1.53, 4.30)	3.74 (2.39, 5.83)	1.26 (0.57, 2.76)
M/L Taper^c^	13,811	1.75 (1.54, 1.99)	2.91 (2.63, 3.23)	1.01 (0.86, 1.18)
M/L Taper Kinectiv	888	2.08 (1.32, 3.28)	4.15 (2.95, 5.81)	1.12 (0.68, 1.85)
Polarstem	2297	1.62 (1.16, 2.25)	2.59 (1.86, 3.59)	1.07 (0.76, 1.52)
Secur-Fit	1618	3.53 (2.72, 4.56)	5.07 (4.04, 6.36)	N/A^b^
Secur-Fit Max	3036	1.93 (1.50, 2.50)	3.25 (2.65, 3.99)	1.09 (0.82, 1.45)
Secur-Fix Plus Max	2120	1.70 (1.23, 2.35)	2.52 (1.93, 3.30)	0.91 (0.67, 1.25)
SROM	1149	1.31 (0.79, 2.17)	3.48 (2.54, 4.75)	N/A^b^
Summit	7009	1.45 (1.19, 1.76)	2.14 (1.81, 2.52)	N/A^b^
Synergy	1374	2.36 (1.68, 3.32)	3.88 (2.94, 5.11)	1.34 (0.95, 1.91)
Taperloc	526	1.52 (0.76, 3.02)	2.50 (1.46, 4.26)	0.94 (0.52, 1.70)
Taperloc 133	12,202	1.79 (1.56, 2.04)	3.00 (2.67, 3.38)	1.05 (0.89, 1.23)
Taperloc 133 Microplasty	4166	1.36 (1.04, 1.77)	2.08 (1.62, 2.67)	0.73 (0.56, 0.94)
Trabecular Metal	1261	1.94 (1.30, 2.87)	2.63 (1.85, 3.72)	1.02 (0.64, 1.61)
Tri-Lock BPS	4142	0.60 (0.41, 0.90)	1.51 (1.12, 2.03)	N/A^b^
Versys Advocate	497	2.13 (1.15, 3.93)	2.62 (1.41, 4.85)	0.92 (0.50, 1.69)
Wagner Cone	628	1.61 (0.87, 2.97)	4.23 (2.76, 6.45)	1.69 (1.11, 2.57)

a indicates zero patients at risk at 5 y following primary procedure.

b indicates the proportional hazards assumption of the Cox model was not met.

c indicates this product does not include M/L Taper Kinectiv.

**Table 3:** Acetabular Cups, 1-year and 5-year CPR with Hazard Ratio (HR) and 95% confidence interval (95% CI).

Cup	Number of cases (N)	1-year CPR (95% CI)	5-year CPR (95% CI)	Implant HR (95% CI)
Continuum	15,041	1.80 (1.60, 2.03)	3.03 (2.75, 3.33)	1.15 (0.99, 1.34)
Converge	532	0.94 (0.39, 2.24)	1.70 (0.89, 3.24)	0.72 (0.37, 1.43)
G7	24,677	1.70 (1.54, 1.87)	2.77 (2.52, 3.05)	N/A^b^
Pinnacle	20,729	0.99 (0.87, 1.14)	1.83 (1.63, 2.05)	0.62 (0.53, 0.73)
PolarCup	570	0.88 (0.37, 2.11)	2.52 (1.40, 4.49)	0.61 (0.23, 1.60)
Procotyl Prime	544	1.77 (0.92, 3.39)	N/A^a^	0.89 (0.46, 1.74)
Reflection	549	1.64 (0.86, 3.13)	2.95 (1.82, 4.78)	0.99 (0.54, 1.81)
Reflection 3	8692	2.11 (1.82, 2.44)	3.43 (3.02, 3.89)	1.36 (1.14, 1.61)
Regenerex RingLoc	1017	1.67 (1.04, 2.68)	2.57 (1.75, 3.75)	0.79 (0.51, 1.23)
RingLoc+	2632	1.75 (1.31, 2.33)	2.63 (2.08, 3.33)	0.84 (0.63, 1.12)
Trabecular Metal	1922	2.05 (1.50, 2.79)	3.82 (3.02, 4.83)	1.32 (0.94, 1.87)
Trident	30,177	1.40 (1.28, 1.54)	2.62 (2.44, 2.82)	N/A^b^
Trident II	13,370	1.64 (1.43, 1.89)	2.15 (1.85, 2.49)	N/A^b^
Trilogy	1908	1.74 (1.24, 2.44)	3.30 (2.57, 4.23)	N/A^b^

a indicates zero patients at risk at 5 y following primary procedure.

b indicates the proportional hazards assumption of the Cox model was not met.

**Figure 2:**
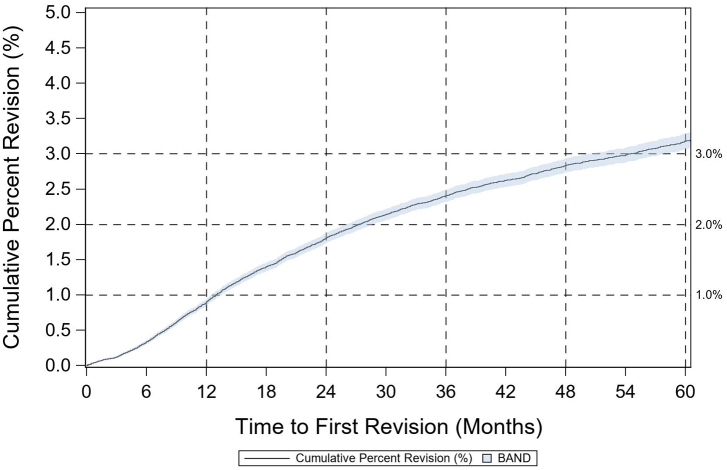
Cumulative percent revision for primary TKA.

There were 245,499 knee arthroplasty cases, of which 92.0% were primary and 8.0% were revision cases. Of these, 206,860, 15,792, and 1350 were primary TKA, unicondylar knee arthroplasty (UKA), and patellofemoral joint (PFJ), respectively. The mean age and BMI of primary TKA cases were 66.4 (SD 9.4) years and 33.3 (SD 6.8), respectively. 62.3% were female. The breakdown of approaches used in primary TKA cases was 86.4% medial parapatellar, 11.3% midvastus vastus medialis obliquus split, 1.3% subvastus, 0.1% lateral parapatellar, and 0.9% other/missing. 86.6% of TKA cases were performed for a diagnosis of osteoarthritis. The 1- and 5-year CPR estimates for primary TKA (Fig. 2) were 0.88% (0.83%-0.92%, 95% CI) and 3.01% (2.93%-3.10%, 95% CI).

As with hips, primary TKA had a wide range of 1- and 5-year revision risks across implants (Table 4). At 1-year, CPRs ranged from 0.20% (0.05%-0.80%, 95% CI) to 2.71% (1.58%-4.63%, 95% CI). At 5-years the values were between 1.10% (0.64%-1.89%, 95% CI) and 12.52% (8.37%-18.50%, 95% CI).

**Table 4:** Total Knee Arthroplasty Components, 1-year and 5-year CPR with Hazard Ratio (HR) and 95% confidence interval (95% CI).

Femur	Tibia	Number of cases (N)	1-year CPR (95% CI)	5-year CPR (95% CI)	Implant HR (95% CI)
Attune	Attune	12,936	0.66 (0.53, 0.83)	3.36 (3.00, 3.76)	N/A^a^
Evolution MP	Evolution MP	3924	1.20 (0.89, 1.62)	4.28 (3.51, 5.21)	1.00 (0.81, 1.25)
Genesis II	Genesis II	1425	1.20 (0.75, 1.92)	3.68 (2.79, 4.85)	1.00 (0.67, 1.50)
Genesis II (CoCr)	Genesis II	730	0.82 (0.37, 1.82)	2.89 (1.85, 4.51)	0.92 (0.51, 1.66)
Genesis II (Oxinium)	Genesis II	695	1.59 (0.88, 2.85)	4.46 (3.14, 6.33)	1.02 (0.64, 1.63)
iBalance	iBalance	539	2.71 (1.58, 4.63)	12.52 (8.37, 18.50)	1.91 (1.23, 2.97)
iTotal		770	1.30 (0.70, 2.40)	4.48 (3.16, 6.34)	1.13 (0.77, 1.68)
iTotal G2+		747	1.34 (0.72, 2.47)	4.11 (2.85, 5.92)	0.98 (0.64, 1.49)
Journey II	Journey	6122	1.41 (1.13, 1.76)	4.58 (3.96, 5.28)	1.35 (1.14, 1.61)
Journey II (Oxinium)	Journey	5354	1.57 (1.25, 1.95)	4.90 (4.24, 5.66)	1.38 (1.16, 1.65)
Journey II BCS (Oxinium)	Journey	841	1.28 (0.69, 2.37)	4.83 (3.35, 6.95)	1.20 (0.83, 1.73)
LCS Complete	M.B.T.	1607	1.35 (0.87, 2.08)	5.04 (3.95, 6.42)	1.40 (0.85, 2.31)
Legion	Genesis II	11,328	1.08 (0.90, 1.29)	3.69 (3.32, 4.11)	1.03 (0.89, 1.19)
NexGen GS	NexGen Pegged	684	1.02 (0.49, 2.13)	2.74 (1.73, 4.33)	1.42 (0.82, 2.47)
NexGen GS	NexGen Precoat	611	0.52 (0.17, 1.60)	1.90 (1.02, 3.51)	0.70 (0.36, 1.36)
NexGen LPS GS	NexGen Precoat	534	0.37 (0.09, 1.49)	2.27 (1.29, 3.96)	0.64 (0.37, 1.09)
NexGen LPS Option	NexGen Precoat	740	0.68 (0.28, 1.62)	2.27 (1.39, 3.68)	0.78 (0.49, 1.22)
NexGen LPS Option	NexGen TM	1298	0.39 (0.16, 0.94)	1.28 (0.77, 2.13)	0.44 (0.22, 0.87)
NexGen Option	NexGen Option	1218	0.33 (0.12, 0.87)	1.10 (0.64, 1.89)	0.61 (0.35, 1.08)
NexGen Option	NexGen Pegged	622	0.80 (0.34, 1.92)	2.89 (1.83, 4.55)	1.64 (0.95, 2.85)
NexGen Precoat	NexGen Precoat	534	0.96 (0.40, 2.28)	2.40 (1.32, 4.32)	0.84 (0.44, 1.60)
NK II	NK II	999	0.20 (0.05, 0.80)	1.41 (0.84, 2.36)	0.54 (0.30, 0.98)
NK II GS	NK II	3693	0.45 (0.28, 0.74)	1.42 (1.04, 1.95)	0.73 (0.47, 1.15)
Persona	Persona	55,225	0.71 (0.64, 0.78)	2.76 (2.60, 2.93)	N/A^a^
Scorpio	Series 7000	654	1.38 (0.72, 2.63)	5.29 (3.78, 7.39)	2.66 (1.56, 4.51)
Sigma	M.B.T.	948	1.52 (0.90, 2.55)	4.50 (3.31, 6.13)	1.10 (0.76, 1.58)
Sigma	Sigma	1603	1.61 (1.09, 2.38)	3.97 (3.09, 5.09)	N/A^a^
Sigma PFC	Sigma	3622	0.68 (0.45, 1.01)	2.34 (1.87, 2.93)	0.67 (0.51, 0.89)
Sigma PFC	Sigma PFC All-Poly	552	0.73 (0.27, 1.92)	1.32 (0.63, 2.74)	1.26 (0.58, 2.76)
Triathlon	Triathlon	34,241	0.88 (0.78, 0.99)	2.52 (2.32, 2.73)	N/A^a^
Triathlon	Triathlon TS	26,258	0.91 (0.80, 1.04)	2.86 (2.63, 3.11)	1.03 (0.91, 1.17)
Vanguard	Maxim	21,870	0.83 (0.71, 0.96)	2.73 (2.51, 2.97)	0.73 (0.64, 0.83)
Vanguard	Maxim Mono-Lock	1200	0.59 (0.28, 1.24)	3.28 (2.31, 4.65)	0.97 (0.69, 1.37)
Vanguard XP	Vanguard XP	547	2.56 (1.52, 4.28)	11.98 (9.47, 15.10)	2.33 (1.75, 3.11)

a Indicates the proportional hazards assumption of the Cox model was not met.

**Figure 3:**
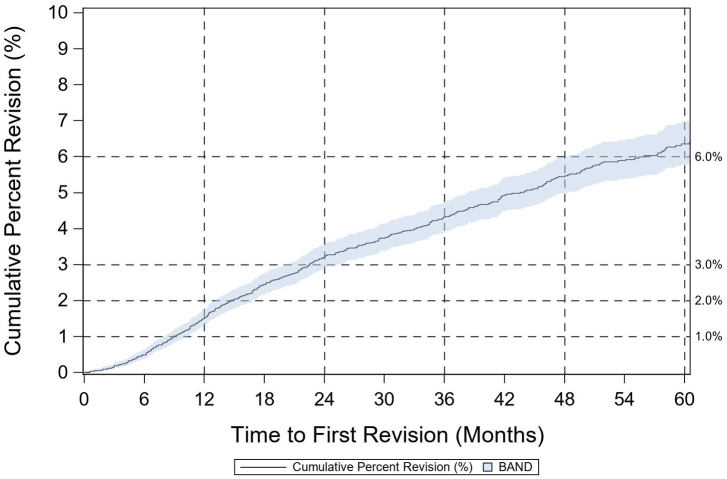
Cumulative percent revision for primary UKA.

The mean age and BMI for UKA cases was 64.1 (SD 10.2) and 31.4 (SD 5.9), respectively, and 50.1% of cases were female. The breakdown of approach was 71.9% medial parapatellar, 20.7% midvastus vastus medialis obliquus split, 3.4% lateral parapatellar, 1.0% subvastus, and 3.0% other/missing. The 1- and 5-year CPR values for primary UKA was 1.41% (1.23%-1.61%, 95% CI) and 5.47% (5.06%-5.90%, 95% CI), respectively (Fig. 3). The lowest 1-year CPR for an implant for primary UKA was 1.08% (0.77%-1.52%, 95% CI) and the highest was 1.79% (1.37%-2.34%, 95% CI) (Table 5). At 5 years, these values were 4.23% (3.54%-5.06%, 95% CI) and 7.13% (6.20%-8.20%, 95% CI).

**Table 5:** Unicompartmental Knee Arthroplasty components, 1-year and 5-year CPR with Hazard Ratio (HR) and 95% confidence interval (95% CI).

Femur	Tibia	Number of cases (N)	1-year CPR (95% CI)	5-year CPR (95% CI)	Implant HR (95% CI)
Oxford	Oxford	3039	1.79 (1.37, 2.34)	7.13 (6.20, 8.20)	1.41 (1.13, 1.76)
Persona	Persona	2110	1.20 (0.80, 1.80)	N/A^a^	N/A^b^
Restoris MCK	Restoris MCK	5799	1.22 (0.96, 1.55)	4.69 (4.05, 5.42)	0.72 (0.56, 0.92)
ZUK	ZUK	3074	1.08 (0.77, 1.52)	4.23 (3.54, 5.06)	N/A^b^

a indicates zero patients at risk at 5 y following primary procedure.

b indicates the proportional hazards assumption of the Cox model was not met.

**Figure 4:**
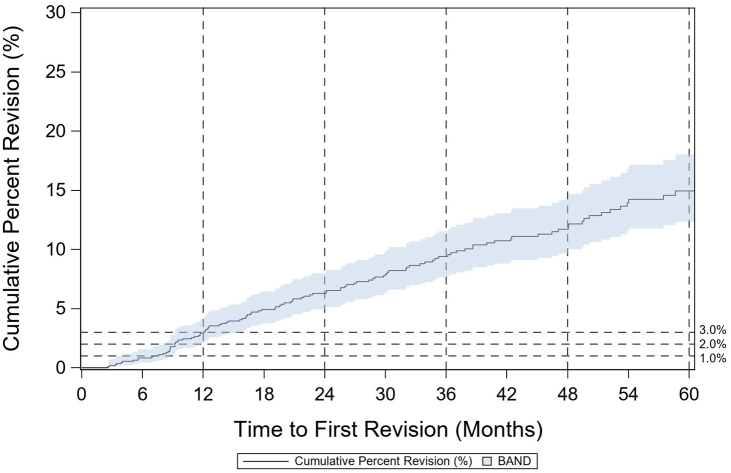
Cumulative percent revision for primary PFJ.

The case volume was insufficient to report product-level revision risks for PFJ implants. The 1- and 5-year CPR estimates for PFJ cases were 2.65% (1.90%-3.70%, 95% CI) and 11.94% (10.10%-14.10%, 95% CI), respectively (Fig. 4).

## Discussion

The objective of this study was to quantify variation in revision risk in THA and TKA by implant using publicly available data in the United States for the full range of adult hip and knee arthroplasty patient ages. There is substantial variability in the 1- and 5-year revision risk across implants. Moreover, there is a strong association between 1- and 5-year revision risks. Previous reports have shown the early survivorship correlates with longer-term outcomes in the Australian Registry, such that no implants that did not achieve an early benchmark went on to achieve a 10-year benchmark [11]. Therefore, surgeons need to consider the wide variability in CPR values reported here and in other registries when selecting implants to improve quality of care.

The primary limitation of this study was that the source of the data was not a nation-wide registry. MARCQI collects data on cases performed in the state of Michigan only. Therefore, these results only provide a regional assessment of implant utilization and performance. Moreover, MARCQI does not capture revision cases that occur outside of Michigan. This may lower the CPR estimates from their actual levels. However, Etkin *et al.* [12] showed that only 4.1% of primary THA/TKA patients migrated out of Michigan within 5 years. Death was not included in the analysis as a competing risk, but Kandala *et al.* [13] showed that including death has a minimal effect on the CPR estimate within 5 years of the primary procedure. Another limitation was using all-cause revision as an endpoint. While the full MARCQI annual report [2] provides the distribution of reasons for revision for each implant, these data were not included here. The interested reader should consult Hughes *et al.* [2]. Moreover, the 5-year follow-up time window was unlikely to reveal effects due to bearing and wear. Because of the possibility of surgeon volume effects, the MARCQI annual report [2] also reports the mean (with standard deviation) and median (with interquartile range) of the number of cases by surgeon and site for each implant. In cases where there may be a concern that the performance of an implant may be impacted by the number of the surgeons using it, the reader should consult the annual report [2]. No information about the distribution of fellowship- vs nonfellowship-trained surgeons in Michigan is available to help interpret these data, which is another limitation.

The primary strength of this study was high coverage rate of knee/hip surgery patients in the database and, most importantly MARCQI’s ability to associate revision cases with primary ones, even if the revision occurs at a different facility and/or by a different surgeon than the primary. This is done through the assignment of patient-specific identification numbers within the MARCQI database along with the utilization of a deterministic hierarchy matching algorithm.

Comparing MARCQI to the AJRR’s reported results [14] is illuminating, although limited by the very different cohorts used and analytical methods used to estimate revision risks. For 5-year revision risk data for THA implants, the 95% CI reported by MARCQI and AJRR did not overlap for 8 implants: Accolade II, Anthology, Corail, Echo Bi-Metric, Polarstem, Secur-Fit, Summit, and Taperloc 133. All of these stems had 95% CI in MARCQI that were completely greater than the corresponding 95% CI in AJRR. That is, the lower limit of the MARCQI CI was greater than the upper limit of the AJRR one. Five cups had nonoverlapping 95% CI: Continuum, G7, Pinnacle, Reflection 3, and Trabecular Metal. Similarly, all have 95% CI in MARCQI that are entirely larger than the AJRR CI. There were 8 stem/cup combinations whose 95% CI did not overlap: Anthology/Reflection 3, Corail/Pinnacle, Echo Bi-Metric/G7, Polarstem/Reflection 3, Secur-Fit/Trident, Synergy/Reflection 3, Taperloc 133/Continuum, and Taperloc 133/G7. As with stems and cups, the MARCQI 95% CI were above the AJRR ones for these stem/cup combinations. In no case were the MARCQI 95% CI entirely less than the AJRR 95% CI. All other stems, cups, and stem/cup combinations were not reported in both the MARCQI and AJRR annual reports and their CI overlapped. Comparisons for TKA implants were not performed because AJRR breaks them down by stability while the 2022 annual report of MARCQI does not. In terms of utilization patterns, it appears that Stryker products are used slightly more in Michigan than the United States, and this may be due to Stryker being located in Michigan. While this is unlikely to affect the CPR estimates, it does make the numbers (N) skewed relative to the AJRR.

The consistency of the pattern between MARCQI and AJRR differences suggests a systemic difference between MARCQI and AJRR. Most likely, MARCQI collects and reports on patients 18 years of age and older, and the AJRR annual report is based on patients 65 years of age and older. Additionally, MARCQI and AJRR use different device libraries, and this could complicate the comparison. For example, MARCQI separates the Corail Coxa Vara from Corail, but AJRR does not list the Corail Coxa Vara at all in its results. It is unclear whether there was an insufficient number of those stems in AJRR or the AJRR library lumps Corail Coxa Vara into Corail. MARCQI scrutinizes the device library it uses to assure meaningful clinical classification, such as splitting out the Corail Coxa Vara. It makes the catalog numbers that comprise each implant product name publicly available online in a supplement to its annual report [15], and it invites input from manufacturers to correct and suggest alternative classification strategies.

These data can be used by surgeons and hospitals, in conjunction with other data sources, to choose implants. Arthroplasty registries in Europe and Oceania have longer follow-up, and summaries have been published by Hughes *et al.* [3] and Foster *et al* [4]. The surgeons and hospitals may identify an implant with a lower rate of revision in the data and consider switching. It is important to remember there is a learning curve for adopting new technology, so it may take many cases to achieve the results reported here and in other registries [16]. This could lead to an increase in revisions before the benefits of the switch are realized. Limiting the number of implant systems used by a surgeon is also critical to achieving and maintaining a low revision rate. The Australian Orthopaedic Association National Joint Replacement Registry has shown that using at most 2 different implant systems for conventional THA is associated with a reduced revision risk [17].

These data can also be valuable to manufacturers in identifying implants with potential concerns. Previously, international registries have been used to call out concerns and have led to the withdrawal of implants from the market. Collaboration between registries and industry is critical to fully understanding and maximizing the value of the data and minimizing harm to patients.

## Conclusions

It is imperative that surgeons track outcomes, especially when switching implants. This can be done by keeping organized records at the practice or hospital level. Ideally, surgeons should participate in a national or regional registry, if available. Since patients often go to other surgeons and hospitals for revision surgeries, the only way to have an accurate assessment of outcomes. Many registries provide surgeon-specific reports that contain funnel plots of revision risk [18] and/or cumulative sum charts [19]. Voluntary registries rely on the participation of surgeons and hospitals to succeed. For example, the AJRR is currently recruiting hospitals to improve the capture of primary and revision cases in the United States. Comprehensive registry coverage with a critical review of the data will enable hospitals, surgeons, manufacturers, and regulators to provide the best outcomes for patients.

## Appendix A Supplementary Data

Conflict of Interest Statement for Hughes

Conflict of Interest Statement for Kim

Conflict of Interest Statement for Hallstrom

Conflict of Interest Statement for Zheng
